# Primary Care Physician Experiences with Integrated Population-Scale Genetic Testing: A Mixed-Methods Assessment

**DOI:** 10.3390/jpm10040165

**Published:** 2020-10-13

**Authors:** Amy A. Lemke, Laura M. Amendola, Kristine Kuchta, Henry M. Dunnenberger, Jennifer Thompson, Christian Johnson, Nadim Ilbawi, Lauren Oshman, Peter J. Hulick

**Affiliations:** 1 Neaman Center for Personalized Medicine, NorthShore University HealthSystem, Evanston, IL 60201, USA; mdunnenberger@northshore.org (H.M.D.); jthompson2@northshore.org (J.T.); cjohnson5@medicine.bsd.uchicago.edu (C.J.); phulick@northshore.org (P.J.H.); 2 Division of Medical Genetics, University of Washington, Seattle, WA 98115, USA; lauraa7@uw.edu; 3 Center for Biomedical Research Informatics, NorthShore University HealthSystem, Evanston, IL 60201, USA; kkuchta@northshore.org; 4 Department of Medicine, University of Chicago, Chicago, IL 60637, USA; 5 Department of Family Medicine, NorthShore University HealthSystem, Evanston, IL 60201, USA; nilbawi@northshore.org (N.I.); loshman@northshore.org (L.O.)

**Keywords:** genomic screening, precision medicine, primary care, clinical implementation, genomics education

## Abstract

The scalable delivery of genomic medicine requires collaboration between genetics and non-genetics providers. Thus, it is essential to investigate and address the perceived value of and barriers to incorporating genetic testing into the clinical practice of primary care providers (PCPs). We used a mixed-methods approach of qualitative interviews and surveys to explore the experience of PCPs involved in the pilot DNA-10K population genetic testing program. Similar to previous research, PCPs reported low confidence with tasks related to ordering, interpreting and managing the results of genetic tests, and identified the need for additional education. PCPs endorsed high levels of utility for patients and their families but noted logistical challenges to incorporating genetic testing into their practice. Overall PCPs were not familiar with the United States’ Genetic Information Nondiscrimination Act and they expressed high levels of concern for patient data privacy and potential insurance discrimination. This PCP feedback led to the development and implementation of several processes to improve the PCP experience with the DNA-10K program. These results contribute to the knowledge base regarding genomic implementation using a mixed provider model and may be beneficial for institutions developing similar clinical programs.

## 1. Introduction

Increased availability and awareness of genetic testing has challenged the traditional clinical model in which genetics professionals serve as the sole provider responsible for ordering and interpreting genetic tests. As of 2019, there were approximately 5 medical geneticists and 12 genetic counselors per million people in the United States [1,2], with the majority of these providers practicing in urban, academic medical centers. The successful, equitable implementation of genomics into mainstream medical care requires scaling genetic service delivery by incorporating non-genetics providers in this process.

Research conducted over the past several years has explored non-genetics providers’ views and perceived barriers related to integrating genomics into their practice. Several studies show that the majority of non-genetics providers report low confidence in genetic concepts, testing and interpretation of results [3,4,5,6,7,8,9,10,11]. High levels of confidence as to when to refer to a genetic specialist have been reported [12], though this was lower in primary care providers (PCPs) than specialty providers. Specifically, non-genetics providers cite challenges related to genomic terminology and the volume of information in genetic laboratory reports [13]. In the context of these knowledge and confidence gaps, specific areas for further education have been identified, including how to interpret results [14] and manage uncertain findings [3]. The value of genetic specialist availability for referral or consultation has been highlighted and a need to clarify the role of non-genetics providers has been suggested [14]. 

Non-genetics providers also report mixed levels of perceived utility of genetic testing [4,14,15,16], which varies based on the adoption of genetics at an institutional level, provider genetic knowledge and provider specialty. A study which incorporated whole genome sequencing into primary care found that these genetic results led to medical recommendations of uncertain clinical utility, as there were no clearly significant short-term impacts on patient care [16]. Further evidence is needed to support the clinical utility of implementing genomic screening at a population level. Despite these challenges, there is early evidence that non-genetics specialty providers and PCPs can successfully and appropriately manage genomic results [5,16], especially in a context where genetics education and individuals with genetics expertise are available. 

To explore the inclusion of non-genetics providers in genetic service delivery, NorthShore University HealthSystem (NorthShore) implemented a combined PCP-genetics provider approach for population genetic testing. This clinical pilot program, the DNA-10K, was launched in 2019. Through this program, patients, regardless of family history, were offered complimentary clinical-grade genetic testing of 60 genes associated with hereditary cancer and cardiac conditions, a 14-gene panel for pharmacogenomics (PGx) testing, ancestry and common trait information (such as lactose intolerance) through Color^TM^. Patients were invited to participate in the clinical program via email and through their NorthShore patient portal. Individuals who agreed to testing consented online in advance of their annual preventative care visit, at which time their PCP could place an order for clinical testing. Over 10,000 patients completed testing in the DNA-10K initiative. 

For the panels related to hereditary cancer and cardiac conditions, pathogenic/likely pathogenic variants and negative results were reported to patients and PCPs. If a variant of uncertain significance (VUS) was detected, a generic statement about VUSs was included on the report. Patients who desired specific details (such as the gene and specific variant) about the VUS were referred to Color^TM^ for more information. Pathogenic/likely pathogenic variants were considered positive results and all other results were labeled as negative and integrated into NorthShore’s electronic health record (EHR). Positive results were imported into the EHR with variant level detail. PCPs were notified of the results via the EHR, and results were available to patients through both Color^TM^’s and NorthShore’s patient portals. All patients with positive results were encouraged and provided with the opportunity to speak with a Color^TM^ genetic counselor. Medical follow-up care related to the test results took place in the NorthShore system.

The purpose of this implementation study was to elicit PCP perceptions of and experiences with incorporating large-scale genetic testing into their clinical practice. The findings aided in evaluation and improvement of program implementation and may more broadly inform best practices for delivering genomic medicine in a mixed primary-care, genetics provider model.

## 2. Materials and Methods

### 2.1. Design

An exploratory sequential mixed-methods design [17] was selected to explore and understand PCP experiences with population genetic testing. In this design, qualitative data are collected and analyzed first. The themes identified are then used to inform the development of a structured survey to quantify the issues identified in the qualitative phase of the research. This study was approved by the NorthShore University HealthSystem IRB (EH19-289).

### 2.2. Qualitative Approach: Semi-Structured Interviews

#### 2.2.1. Recruitment

Study participants were NorthShore PCPs practicing at one of the 14 sites offering the DNA-10K program. A purposive sampling plan included identification of PCPs from a range of practice locations and primary care specialties, including internal medicine, family medicine and obstetrics/gynecology (OB/GYN). To be eligible for the study, PCPs needed to have at least five patients who received genetic results from the DNA-10K. PCPs received an email invitation to participate in a research interview and two follow-up reminders. Interview participants were compensated with a USD 100 service award.

#### 2.2.2. Data Collection

Qualitative semi-structured interviews were conducted to allow for discovery of unanticipated findings, clarification of viewpoints and as a strategy to learn more about PCP barriers and facilitators in utilizing population genetic testing in their practice. Two interviewers trained in qualitative research (JT, CJ) used an interview guide that contained eight open-ended questions that directly related to the study aims (Supplementary Materials S1). The interview guide was pretested through five cognitive interviews with PCPs practicing in the NorthShore primary care network [18]. Revisions were made based on feedback from the cognitive interviews and from the multidisciplinary study team. Interviews were conducted and analyzed on an ongoing basis until thematic saturation was reached, which occurs when no new information is observed.

#### 2.2.3. Data Analysis

Recordings of interviews were transcribed verbatim and independent checks by two investigators confirmed accurate transcription. Atlas.ti, a qualitative software program, was used to organize and manage the data [19]. The interview guide was first used to develop a list of provisional codes. The codes were then refined and changed as new ideas were encountered in the reading of each new transcript. A final codebook of 16 codes, with definitions and examples, was used by the investigators to identify key opinions and themes. Two investigators double-coded a subset (6, 35%) of the transcripts to assess consistency in code assignment. The team worked collaboratively to reach coding agreement and any final discrepancies defaulted to a senior coder. Grounded theory was used as a general guide to allow themes and theory to emerge from the transcript data [20]. The themes were collectively explored and interpreted by the research team. Data reduction and analysis were conducted through summative content analysis [21] with the aim of describing the participants’ views toward and experiences with population genetic testing in their practices. Key quotes that highlighted the main themes and sub-themes of the interview findings were identified by the investigators.

### 2.3. Quantitative Approach: Survey

#### 2.3.1. Recruitment

All NorthShore PCPs participating in the DNA-10K who had patients with results returned were invited by email to participate in the online research survey. Two follow-up email reminders were also sent. PCPs who responded received a USD 10 Panera gift card for completing the survey.

#### 2.3.2. Data Collection

The Tailored Survey Design method was used as a general guide in survey development and format [22]. Findings from the qualitative interviews informed question inclusion and categorical responses used in the survey. Five cognitive interviews were conducted with PCPs similar to those PCPs invited to respond to the survey to increase readability and validity of the tool [18]. A number of survey questions were utilized or adapted, with permission, from unpublished survey items from the Electronic Medical Records and Genomics (eMERGE) consortium, the Clinical Sequencing Evidence-Generating Research (CSER) consortium and Ingrid Holm, PI. Likert scales were used in the majority of the response categories. The survey email invitations contained a link to a REDCap [23,24] 39-item online survey (Supplementary Materials S2). Thirty-two physicians requested paper-based surveys, which were made available to them.

#### 2.3.3. Data Analysis

Survey results were summarized using frequencies and percentages. Because participants were allowed to skip individual items, the sample size varied by question. Throughout the paper, percentages reported reflect the valid percent (excludes missing answers). For questions using the five-point Likert scale response (strongly disagree, somewhat disagree, neither agree nor disagree, somewhat agree and strongly agree) and the four-point response scale (not at all, very little, somewhat and to a great extent), data were first summarized and reviewed in their original form and then collapsed to two categories to facilitate analysis and interpretation. Comparisons between demographic factors were assessed using two-tailed chi-square or Fisher’s exact (for small sample size) tests. Statistical significance was defined as *p* < 0.05. All statistical analysis was performed using SAS 9.3 [25]. 

## 3. Results

### 3.1. Qualitative Results

Audiotaped telephone interviews were conducted from October to December 2019. Interviews lasted on average 30 min and saturation was reached after 17 interviews, which falls within the reported range for this type of qualitative approach [26].

#### 3.1.1. Participant Characteristics

Approximately 70% of the 17 PCPs participating in the interview portion of the study were female (n = 12). In total, 8 practitioners were in internal medicine, 5 were in family medicine and 4 were in OB/GYN. In total, 10 of the 14 practice sites were represented.

#### 3.1.2. Semi-Structured Interviews: Major Themes

Three broad themes emerged regarding PCP experiences with the DNA-10K population genomics testing program: benefits to clinical care provision, challenges in practice and recommended improvements. These themes and sub-themes are presented, along with exemplary quotes from the PCP interviews, in Table 1. 

Overall, PCPs highlighted the value of genetic testing in identifying risk to detect and prevent disease in patients and their families. Major challenges related to patient privacy concerns, prioritizing genetic discussions amongst other preventive care and a lack of knowledge and skill discussing positive results. Recommended improvements suggested by PCPs included the need for both patient and provider educational resources. 

### 3.2. Quantitative Results

Surveys were collected from February to April 2020. Seventy of the 104 eligible PCPs responded to the survey for a response rate of 67.3%, and the mean length of time to complete the online survey was 6.5 min. PCPs from all 14 practice sites responded to the survey. 

#### 3.2.1. Participant Characteristics 

The characteristics of the 70 PCPs who responded to the survey are presented in Table 2. Half of the 70 PCPs were female (51%, N = 35); most PCPs identified as White (80%, N = 52) and had greater than 10 years of experience in clinical practice (71%, N = 50). The most common primary area of practice was internal medicine (59%, N = 41) and the majority of participants (96%, N = 67) spent greater than 50% of their time in patient care-related activities. PCPs had varying numbers of their patients participating in the DNA-10K, with a median of 84.5 (IQR: 46–145) and range of 1–273 patients per physician.

#### 3.2.2. Survey Findings

##### Benefits of Genetic Testing

The utility of the genetic test result was endorsed by PCPs across several survey questions. Seventy-seven percent of PCPs somewhat or strongly agreed that the genetic testing program is useful to change their current management of patients’ care, and most PCPs agreed that the genetic testing program has value in identifying the need for increased disease screening (81.4%) and supporting management of patients’ care that is already underway (69.6%). Most PCPs (81.4%) also agreed that there is value in identifying at-risk family members. PCPs reported recommending patients’ family members undergo genetic testing most commonly for cancer risk results (61.4%), followed by cardiac risk results (21.7%) and PGx results (8.8%). 

##### Challenges in Practice 

**Figure 1:**
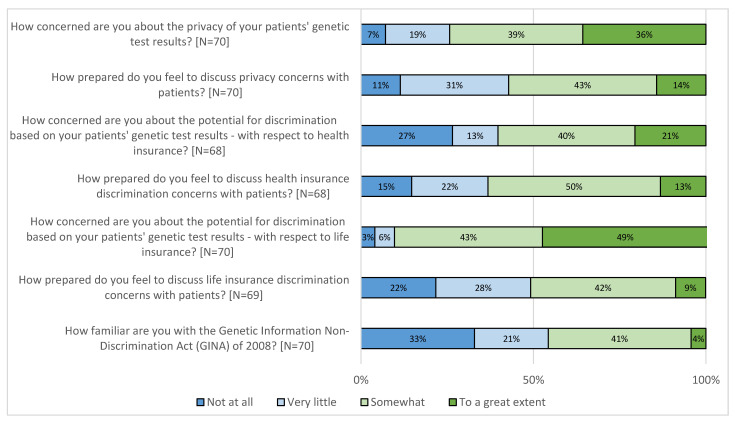
Primary care providers’ (PCP) concern about and preparedness to discuss privacy and insurance discrimination.

PCP concerns for patient privacy and genetic discrimination, as well as a lack of awareness of the United States’ Genetic Information Nondiscrimination Act (GINA) of 2008, were highlighted in the survey data (Figure 1). Most PCPs (74.3%) reported feeling concerned about the privacy of their patients’ genetic test results and the potential for health (60.3%) and life (91.5%) insurance discrimination. A small number (4.3%) of respondents reported being familiar to a great extent with the GINA, and approximately a third (32.9%) reported knowing nothing at all about it. However, more than half of PCPs felt somewhat or to a great extent prepared to discuss these topics (privacy 57.2%; health insurance discrimination 63.2%; life insurance discrimination 50.7%). 

PCPs reported varying degrees of confidence with their knowledge and skills across the genetic testing process. Approximately half (52.8%) of PCPs agreed that they feel confident explaining the risks and benefits of genetic testing to their patients, and less than half somewhat or strongly agreed they feel confident in their ability to explain genetic test results related to cancer risk (42.9%), cardiac risk (27.2%) and PGx (32.8%). PCPs’ reported confidence in explaining results was slightly higher than their reported ability to articulate clear next steps for the different categories of results (cancer risk 51.4%; cardiac risk 42.0%; PGx result 34.8%). 

Most PCPs (86.8%) reported that the genetic testing program has increased their workload, and approximately a third (37.3%) either somewhat or strongly agreed that this increase was reasonable. However, more than half (56.5%) were satisfied overall with the DNA-10K program, and the majority reported satisfaction with the workflow for offering (61.4%) and ordering (70.0%) genetic testing. Comparatively, PCPs reported lower satisfaction with the process by which patients (29.8%) and providers (42.0%) receive test results. 

##### Recommendations for Additional Education 

**Figure 2:**
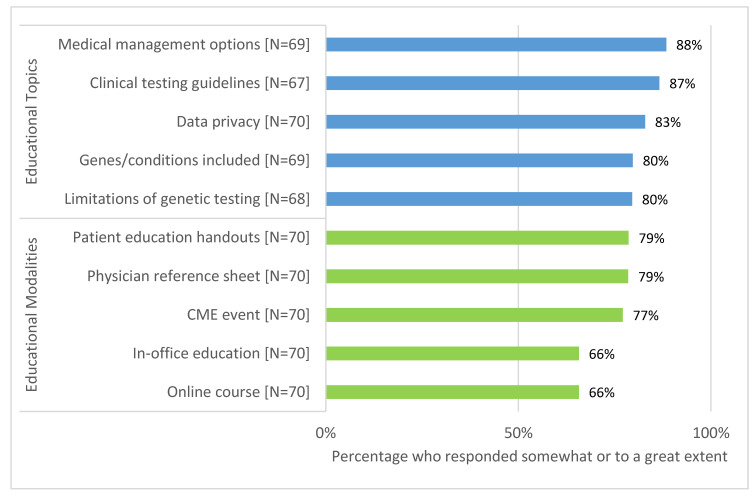
Requested education topics and preferred modalities.

Over one quarter of PCPs (28.9%) somewhat or strongly agreed that they have received adequate training to offer genetic testing in their practice. Less than half (40.0%) reported being somewhat or very confident in their knowledge of genetics, their ability to explain genetic concepts (47.1%) and results (34.8%) to patients and their ability to respond to patient questions about genetic technologies (27.9%). Additional education about medical management options for patients with a positive result (88.4%) and clinical testing guidelines (86.6%) were most frequently requested, and the majority of PCPs endorsed the value of patient education handouts (78.6%) and physician reference sheets (78.5%). Preferred educational topics and modalities regarding genetic testing are summarized in Figure 2. 

#### 3.2.3. Exploration of Survey Results by Demographic Characteristics

The age of PCPs influenced how likely they were to report feeling confident and prepared for several aspects of the genetic testing process. PCPs aged 50 or greater were more likely to report they were not at all confident in their ability to explain a genetic test result compared to those less than 50 years old (44.8% vs. 23.1%, *p* = 0.044). However, PCPs aged 50 or older were more likely to feel somewhat or to a great extent prepared to discuss privacy concerns (79.3% vs. 42.5%, *p* = 0.004) and health insurance discrimination (82.1% vs. 48.7%, *p* = 0.006). PCPs who did not identify as White were more likely to report higher levels of concern for privacy of patients’ genetic test results (92.3% vs. 69.2%, *p* = 0.023). With regard to utility of testing, internal medicine PCPs were more likely to somewhat or strongly agree that the genetic testing program is useful to change their current management of patients’ care than family medicine or OB/GYN providers (87.1% vs. 62.1%, *p* = 0.038). 

### 3.3. Post-Study Program Modifications

**Figure 3:**
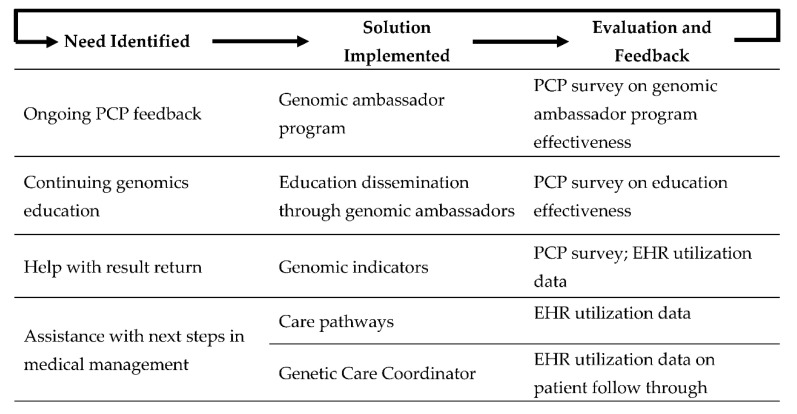
Continuous process improvement plan.

In response to the identified need for process improvement and additional education, we made a number of programmatic changes related to patient clinical outcomes, general genetic education and operational details (Figure 3). As a first step, increased PCP input was identified as an ongoing need for the program. To encourage ongoing feedback, we created the genomic ambassador program, comprised of 12 PCPs involved in the DNA-10K. This group meets quarterly to review workflow processes and provide programmatic feedback. These PCPs gave input on how results would appear in the EHR and how clinical decision alerts would fire to inform PCPs about genetic testing results. In response to the request for continuing genetics education, the genomic ambassadors are provided a quarterly online genomics education curriculum and application, which they can share with their clinic staff and PCP network partners. These brief educational sessions focus on workflow processes and basic science medical genetics education. An evaluation of the educational program is ongoing.

In response to the identified need to support PCPs with the return of genetic results and next steps in medical management, a section called “genomic indicators” was enabled in the EHR. Genomic indicators are groupings based on the gene and the reported pathogenicity of the variant. For example, *BRCA1* c.4327C > T (rs41293455) is assigned to the genomic indicator as “*BRCA1* pathogenic variant”. Each genomic indicator is presented to providers with a clinical explanation and recommendations for next steps. Additionally, genomic indicators provide information used to trigger other clinical decision support tools in the EHR, such as genetic care pathways. A care pathway is intended to track a patient’s progress through the recommended enhanced screening protocol based on a positive genetic result. To date, one care pathway has been implemented and three additional care pathways are in development. By tracking patients’ progress, the Center for Personalized Medicine is able to provide continued support and oversight of patients’ medical management, and it is able to identify areas in need of further process improvement to ensure our patients are receiving appropriate care based on their genetic results. Finally, to further assist PCPs in providing appropriate medical management to patients with positive results, and to more seamlessly transfer patients from primary care to specialty care, we are piloting the Genetic Care Coordinator (GCCs) role. GCCs are responsible for guiding patients through their screening and risk reduction activities after a positive genetic result. EHR utilization data will be used to assess the impact of these interventions. 

## 4. Discussion

The participation of PCPs as key stakeholders in a primary care-based genetic screening program is imperative for scaling the safe and effective delivery of precision medicine [27]. Here we present the results of a mixed-methods study exploring PCPs’ experiences implementing the DNA-10K population genetic testing pilot program. 

Previous research has identified mixed levels of utility endorsed by PCPs with respect to genetic and genomic testing [4,11,14,15,16]. The majority (between 70% and 81%) of surveyed PCPs in the present study agreed that the genetic results would impact and support current patient management and had value in identifying patients and their family members with an increased need for screening. These findings are higher than other available studies which report a range from 32% to 67% of PCPs finding genetic testing useful in a variety of settings [4,15]. The relatively high level of perceived value of genetic testing in this study may have been influenced by provider’s experiences in the program and/or the integration of genetics at the institutional level. This is supported by Lerner et al.’s finding that an institutional culture which fosters adoption of genomic medicine is associated with higher stakeholder valuation of genetic testing [15]. Nonetheless, it is imperative for PCPs to agree with the utility of genomics in a mixed-provider model of its integration into patient care.

Similar to existing literature [5,6,10,14,28,29], PCPs participating in this study also expressed concerns about patient privacy and potential discrimination related to patient genomic test results. Concern regarding privacy and health or insurance discrimination was noted during PCP interviews and was endorsed by most surveyed PCPs. Of note, PCPs were considerably more concerned about privacy and insurance discrimination than surveyed patients who had clinical testing through the DNA-10K [30]: privacy (PCPs: 74.3% vs. patients: 47%); health insurance (60.3% vs. 38%); life insurance (91.5% vs. 36%). PCP concern about privacy and discrimination has the potential to be a barrier to patient referral or completion of genetic services and testing [10,29], thus PCP awareness of this discrepancy in concern levels is important in order to ensure the equitable offering of services to all patients. The majority of surveyed PCPs reported little to no familiarity with the GINA. Increased awareness of the GINA could help address the lack of preparedness felt by many PCPs, especially those with less clinical experience. Other countries—including Australia, Canada, the United Kingdom and several others in the European Union—also have laws to protect against various aspects of genetic discrimination [31], and therefore the need to increase provider knowledge of privacy and genetic discrimination laws may be broadly applicable.

Several of our findings are consistent with previous studies exploring PCP perspectives towards incorporating genomic medicine into their practice. Previous qualitative and quantitative studies have identified a lack of PCP confidence in their genetics knowledge as a barrier to incorporating genetics into their practices [3,4,5,6,7,8,9,10]. These studies report PCP confidence in their knowledge of various aspects of genomics to be between 15% and 50% [3,4,5,6,7,9]. Similarly, in the present study, only half of PCPs surveyed about the DNA-10K reported confidence in discussing the risks and benefits of testing, explaining results and articulating next steps to their patients. Additionally, PCPs reported challenges surrounding interpreting and discussing results, as well as uncertainty for next steps after results are returned. The majority of PCPs desired additional education and resources on these topics, likely due to their reported lack of confidence. These findings add to the existing literature which indicates PCPs need further education in order to successfully integrate genetics into their clinical practice [3,5,6,7,8,9,10,14]. It is likely that effective genomic education will need to be tailored to individual provider learning preferences and by specialty [32] and/or be made available to PCPs at the point of care.

Previous studies have also identified barriers to incorporation of genomic testing into clinical practice by PCPs related to logistical or health system-level issues [9,10]. Most surveyed PCPs involved in the DNA-10K program reported that their workload increased as a result of participating in the initiative. Interviewed PCPs also highlighted limited time and competing interests in practice, which unfortunately, are common issues faced by PCPs [10,33]. PCPs also echoed previous findings [10,14] that they felt unclear about their role, particularly in results disclosure. Creating efficient workflows with adequate support for PCPs, and defining clear roles for PCPs and genetics specialists, is essential for a sustainable model to deliver large scale genetic testing at an institutional level.

Using a learning healthcare system model [34], we have been able to identify the needs of key stakeholders and develop potential solutions for programmatic improvement. Previous PCP feedback elicited through formal assessments of other genomic initiatives at our institution has been key in informing processes, workflows and educational efforts implemented in the DNA-10K [35,36]. Moving forward, it will be important to continually elicit feedback from PCP genomic ambassadors and other stakeholders so we can continue to iterate and improve both the user experience and accessibility of personalized medicine to all patients.

### Study Strengths and Limitations

This study explores several factors involved in the practice of genomic medicine by PCPs, and our findings both support and add to previous research in this area [32]. The mixed-methods approach used in this study combines the benefits of both interview and survey data enabling a deeper understanding of the survey responses and integration of results to strengthen the conclusions. Our qualitative interview findings add validity to the types of questions included in the survey, and the survey enabled quantification of the degree to which physicians felt about the items brought up in the interviews. However, the findings from this study have limitations. The experience of PCPs who did not respond to the survey or take part in qualitative interviews may have been different from those who participated. Furthermore, the perspectives of surveyed and interviewed PCPs were elicited in the context of the DNA-10K initiative, so some findings may be specific to this clinical program and patient population. There may be a selection bias in the DNA-10K program if the patients who took part are not representative of a broader patient population in terms of education level, race and gender. For example, physician experiences with a highly educated patient population taking part in genetic testing programs may be different from the experiences of physicians with patients having a lower literacy level.

## 5. Conclusions

PCPs involved in the DNA-10K endorsed high levels of utility for genetic testing but cited logistical challenges with implementing the program into their clinical practice. Similar to previous research in non-genetics providers, PCPs involved in the DNA-10K also reported low confidence in this context and a need for additional genomics education and resources. Findings from this mixed-methods research were key to the development of several interventions to support PCPs and ongoing implementation efforts. As PCPs are the foundation of preventive medicine, it is important they be engaged in the integration process in order to realize the potential of genomics in health care and reduce risk for disease. Findings from this study and their implications for the DNA-10K program may be beneficial for other institutions undertaking the challenge of clinical implementation of genomics-guided care across their health system. Future research is needed to examine PCPs’ perspectives and experiences with population genetic testing in diverse patient populations and in a variety of healthcare settings.

## Figures and Tables

**Table 1:** Themes and illustrative quotes from primary care physician interviews.

Themes and Sub-Themes	Illustrative Interview Quotes
Benefits to clinical care provision	
Detect/prevent disease	“The benefits are that we discover something on genetic testing that sheds light on increased risk or something we are not screening for; might trigger additional screening.” (P5)
Increase access to genetic services	“For some reason it was really hard to get people to genetics, even when they had compelling family histories; …there’s a lower barrier with integrated testing.” (P9)
Identify patients at risk with no family history of disease	“I have had some patients, adopted and those not knowing their family histories, and one came back as Lynch positive. So changed everything I counseled and recommended.” (P10)
Improve medication management based on pharmacogenomics results	“I have had a number of patients who are on a lot of antidepressants and we’ve been able to fine tune their medication regimes as a result of testing.” (P7)
Family implications	“Then also to know for children, so that you know if kids need to be screened for anything as well.” (P3)
Challenges in practice	
Privacy and insurance concerns	“There are some people out there who really worry about the idea of their DNA being tested…is somebody going to use it against them, like for health/life insurance.” (P9)
Time constraints and competing priorities	“There’s such a big list of things that we need to review and talk about and recommend to patients, that it becomes a challenge for time having yet another thing to talk about.” (P5)
Interpreting and discussing results	“Having potentially to field questions from patients, where I don’t have the skillset to tell them. A negative result is pretty easy, but for positive results I don’t have the full skillset.” (P15)
Unclear about next steps for medical management	“Sometimes there are results we are unfamiliar with or unprepared to deal with, and not sure about where to direct patients to go and what surveillance is appropriate.” (P7)
Role in results disclosure workflow unclear	“I can’t tell like, did somebody call the patient, am I supposed to be doing something? I can’t see anything in the chart to know if someone got a hold of the patient with results.” (P14)
Recommended Improvements	
Patient education resources	“I think brochures are key because the patient can actually hold onto it and take a look as opposed to just on electronic format.” (P12)
Physician education	“I think there are still physicians who don’t buy in because they feel like this is not part of their primary care field. And they don’t understand the genes being tested. We need to provide easy access to CME.” (P6)
Additional resources	“Some sort of reference, even if it was just literally a PDF that people could download on their own.” (P2)

**Table 2:** Characteristics of survey participants.

	N (%)
Total Respondents	70
Age group: [N = 69]	
20–29	0 (0.0)
30–39	17 (24.6)
40–49	23 (33.3)
50–59	18 (26.1)
60–69	8 (11.6)
70 or older	3 (4.4)
Gender: [N = 69]	
Male	34 (49.3)
Female	35 (50.7)
Non-binary	0 (0.0)
Other	0 (0.0)
Race/Ethnicity: [N = 65]	
American Indian or Alaska Native	0 (0.0)
Asian	12 (18.5)
Black or African American	0 (0.0)
Hispanic or Latino	0 (0.0)
Native Hawaiian or Other Pacific Islander	1 (1.5)
White	52 (80.0)
Primary area of practice: [N = 70]	
Internal Medicine	41 (58.6)
Family Medicine	18 (25.7)
Obstetrics/Gynecology	11 (15.7)
Other	0 (0.0)
How many years have you been in clinical practice? [N = 70]	
0–5	10 (14.3)
6–10	10 (14.3)
11–15	8 (11.4)
16–20	18 (25.7)
21 or more	24 (34.3)
Percentage of time is in patient care-related activities: [N = 69]	
0–30	0 (0.0)
31–40	2 (2.9)
41–50	1 (1.4)
51 or more	67 (95.7)

## Supplementary Materials

The following are available online at https://www.mdpi.com/2075-4426/10/4/165/s1, Supplementary Materials S1: Semi-structured interview guide, Supplementary Materials S2: Physician survey.
