# Supplementary material for: Primary Care Physician Experiences with Integrated Population-Scale Genetic Testing: A Mixed-Methods Assessment
Source: J Pers Med. 2020 Oct 13;10(4):165. doi: 10.3390/jpm10040165 (PMC7720124; doi:10.3390/jpm10040165)
Supplement: Supplementary file 1 [file jpm-10-00165-s001.zip › Lemke et al_EH19-289_Supplementary Mat 1_Interview guide.pdf]

Primary Care Physician Experiences with Pre-emptive Genetic Testing (DNA-10K) in an Integrated Health System

Thank you for participating in NorthShore's study on pre-emptive genetic testing (DNA-10K). In this initiative, genetic testing is done in ostensibly healthy patients and provides information about their risk to develop certain diseases. Findings from this research will help us address the needs of primary care physicians and prepare for the integration of population genetic testing at NorthShore University HealthSystem. Please note that all responses will be kept strictly confidential and no individual responses will be shared. This recorded interview should take approximately 30 minutes to complete. We appreciate your time. Do you have any questions before we begin? Do you agree to proceed with participating in this interview?

Interview Guide

1. What has your experience been like with implementing pre-emptive genetic testing (DNA-10K) in your practice?
   Probe: Overall, how satisfied are you with the offering of pre-emptive genetic testing (DNA-10K) in your practice?

2. What are some potential benefits of offering pre-emptive genetic testing (DNA-10K) to patients?
   Probe: Please describe your views on the value, or utility, of this type of testing.
   Probe: Do you have any patient examples to share?

3. What are some potential challenges of offering pre-emptive genetic testing (DNA-10K) to patients?
   Probe: Do you have any patient examples to share?

4. How prepared do you feel in discussing pre-emptive genetic testing (DNA-10K) as an option with patients?
   Probe: What might be some ways to help you become more prepared?

5. Logistically, how has the workflow for offering the DNA-10K been working in your practice?
   Probe: What might be some ways to improve this process?
   Probe: Any changes you would like to recommend?

6. What has your experience been like in discussing pre-emptive genetic testing ***results*** with patients? *(this might include results related to cancer, cardiology, pharmacogenomics, and non-medical genetic traits like ancestry, etc.)*
   Probe: Do you have any patient examples to share?
   Probe: How prepared do you feel in discussing results with patients?
   Probe: What might be some ways to help you become more prepared?

7. What kinds of resources would be most helpful to you in implementing pre-emptive genetic testing (DNA-10K) in your practice?

8. What might be some reasons you think patients might not follow-through with a blood draw after consenting to participate in the DNA-10K?

9. Do you have any other comments to share?
