# Supplementary material for: Primary Care Physician Experiences with Integrated Population-Scale Genetic Testing: A Mixed-Methods Assessment
Source: J Pers Med. 2020 Oct 13;10(4):165. doi: 10.3390/jpm10040165 (PMC7720124; doi:10.3390/jpm10040165)
Supplement: Supplementary file 1 [file jpm-10-00165-s001.zip › Lemke et al_EH19-289_Supplementary Mat 2_PCP Survey.pdf]

RESEARCH SURVEY

Primary Care Provider Experiences with Genetic Testing (DNA-10K) in an Integrated Health System

Thank you for agreeing to participate in our study on pre-emptive genetic testing (DNA-10K) in primary care. *Pre-emptive* testing refers to genetic testing offered to patients, regardless of family history, at the time of their annual physical exam. This complimentary testing (Color panel including cancer, cardiac, and pharmacogenomics results) was offered from April 2019 to January 2020 - through primary care. Findings from this survey research will help us address the needs of primary care physicians and prepare for the integration of large-scale genetic testing.

Survey Questions

Please select the best response for each question below. There is a comment box at the end of the survey, if you would like to provide additional information or feedback.

Please CIRCLE your response (select one option) to the questions below.

<u>DNA-10K Patient Results</u>

For each of your patients who participated in the DNA-10K genetic testing program, you received results related to ***cancer risk, cardiac risk, and pharmacogenomics****. The following questions ask you about each type of result from the DNA-10K.*

Cancer Risk Results (DNA-10K)

1. Approximately how many patients have you discussed genetic testing **cancer risk** results with?

None

1-5

6-10

11-15

16-20

>20

Over →

2. I feel confident in my ability to explain genetic test results to my patients related to **cancer risk.**

Strongly disagree

Somewhat disagree

Neither agree nor disagree

Somewhat agree

Strongly agree

3. I feel confident in my ability to articulate clear next steps (*referrals, additional testing, etc*.) to my patients after they receive a *positive* **cancer risk** result.

Strongly disagree

Somewhat disagree

Neither agree nor disagree

Somewhat agree

Strongly agree

4. Have you recommended patients' family members undergo genetic testing, based on your patients' **cancer risk** test results?

Yes

No

Cardiac Risk Results (DNA-10K)

5. Approximately how many patients have you discussed genetic testing **cardiac risk** results with?

None

1-5

6-10

11-15

16-20

>20

Over →

6. I feel confident in my ability to explain genetic test results to my patients related to **cardiac risk.**

Strongly disagree

Somewhat disagree

Neither agree nor disagree

Somewhat agree

Strongly agree

7. I feel confident in my ability to articulate clear next steps *(referrals, additional testing, etc.)* to my patients after they receive a *positive* **cardiac risk** result.

Strongly disagree

Somewhat disagree

Neither agree nor disagree

Somewhat agree

Strongly agree

8. Have you recommended patients' family members undergo genetic testing, based on your patients' **cardiac risk** test results?

Yes

No

Pharmacogenomics Result (DNA-10K)

9. Approximately how many patients have you discussed **pharmacogenomics** results with?

None

1-5

6-10

11-15

16-20

>20

Over →

10. I feel confident in my ability to explain **pharmacogenomics** results to my patients**.**

Strongly disagree

Somewhat disagree

Neither agree nor disagree

Somewhat agree

Strongly agree

In the next question, "actionable" refers to pharmacogenomics results which may influence your clinical decision process when selecting a medication or dose for a patient (i.e. by identifying patients at greater risk of side effects or therapeutic failure).

11. I feel confident in my ability articulate clear next steps to my patients after they receive an *actionable* **pharmacogenomics** result.

Strongly disagree

Somewhat disagree

Neither agree nor disagree

Somewhat agree

Strongly agree

12. Have you recommended patients' family members undergo genetic testing, based on your patients' **pharmacogenomics** results?

Yes

No

Over →

Genetic Testing Workflow

The DNA-10K testing workflow involves a number of components including offering testing, ordering testing, and returning results to patients.

Offering *the genetic testing refers to the online patient message and consent process prior to DNA-10K testing.*

13. I am satisfied with the workflow for *offering* genetic testing to my patients.

Strongly disagree

Somewhat disagree

Neither agree nor disagree

Somewhat agree

Strongly agree

Ordering *the DNA-10K genetic test refers to the physician process of placing the order in Epic.*

14. I am satisfied with the workflow for *ordering* genetic testing for my patients.

Strongly disagree

Somewhat disagree

Neither agree nor disagree

Somewhat agree

Strongly agree

The following two questions ask about your experience with the ***results return process****.*

15. I am satisfied with how I am informed of my patients' DNA-10K results in Epic.

Strongly disagree

Somewhat disagree

Neither agree nor disagree

Somewhat agree

Strongly agree

Over →

16. I am satisfied with how my patients are informed of their results.

Strongly disagree

Somewhat disagree

Neither agree nor disagree

Somewhat agree

Strongly agree

17. The DNA-10K genetic testing program has increased my workload.

Yes

No (skip to Q 19)

18. The additional workload due to the DNA-10K seems reasonable.

Strongly disagree

Somewhat disagree

Neither agree nor disagree

Somewhat agree

Strongly agree

<u>DNA-10K Value and Satisfaction</u>

*The following five items relate to the **clinical utility** of genetic testing in the context of the pre-emptive DNA-10K test offering.*

19. I believe genetic testing is useful to:

a. Change current management of patients' care

Strongly disagree

Somewhat disagree

Neither agree nor disagree

Somewhat agree

Strongly agree

Over →

19. I believe genetic testing is useful to:

b. Support the management of patients' care already underway

Strongly disagree

Somewhat disagree

Neither agree nor disagree

Somewhat agree

Strongly agree

c. Identify need for increased disease screening

Strongly disagree

Somewhat disagree

Neither agree nor disagree

Somewhat agree

Strongly agree

d. Identify at-risk family members

Strongly disagree

Somewhat disagree

Neither agree nor disagree

Somewhat agree

Strongly agree

e. Provide information for adopted individuals

Strongly disagree

Somewhat disagree

Neither agree nor disagree

Somewhat agree

Strongly agree

Over →

20. Overall, I am satisfied with the DNA-10K genetic testing program.

Strongly disagree

Somewhat disagree

Neither agree nor disagree

Somewhat agree

Strongly agree

DNA-10K Preparedness

21. I received adequate training to offer genetic testing to patients in my practice.

Strongly disagree

Somewhat disagree

Neither agree nor disagree

Somewhat agree

Strongly agree

Not applicable

22. Overall, I feel confident when explaining the risks and benefits of genetic testing to my patients.

Strongly disagree

Somewhat disagree

Neither agree nor disagree

Somewhat agree

Strongly agree

Over →

23. How confident are you…

a. In your knowledge about genetics?

Not at all confident

A little confident

Somewhat confident

Very confident

b. In your ability to explain genetic concepts to patients?

Not at all confident

A little confident

Somewhat confident

Very confident

c. In your ability to respond to patients' questions about genetic testing technologies?

Not at all confident

A little confident

Somewhat confident

Very confident

d. In your ability to explain a result from a genetic test to patients?

Not at all confident

A little confident

Somewhat confident

Very confident

Over →

Preferred Educational Topics and Modalities

Please indicate the degree to which you are interested in learning more about the following topics related to providing genetic testing.

24a. Genes/conditions included on test

Not at all

Very little

Somewhat

To a great extent

b. Clinical testing guidelines

Not at all

Very little

Somewhat

To a great extent

c. Medical management options for patients with a positive result

Not at all

Very little

Somewhat

To a great extent

d. Limitations of genetic testing

Not at all

Very little

Somewhat

To a great extent

Over →

Please indicate the degree to which you are interested in learning more about the following topics related to providing genetic testing.

e. Data privacy

Not at all

Very little

Somewhat

To a great extent

f. Other topics *(please specify):*

A number of modalities in which genetic testing education can be provided are listed below.

Please indicate the degree to which you prefer the following education modalities in learning about topics related to genetic testing.

25a. CME Event

Not at all

Very little

Somewhat

To a great extent

b. In-office education

Not at all

Very little

Somewhat

To a great extent

Over →

Please indicate the degree to which you prefer the following education modalities in learning about topics related to genetic testing.

c. Online course

Not at all

Very little

Somewhat

To a great extent

d. Physician reference sheet

Not at all

Very little

Somewhat

To a great extent

e. Patient education handouts

Not at all

Very little

Somewhat

To a great extent

Data Privacy

The following questions ask about privacy and insurance issues.

26. How concerned are you about the privacy of your patients' genetic test results?

Not at all

Very little

Somewhat

To a great extent

Over →

27. How prepared do you feel to discuss privacy concerns with patients?

Not at all

Very little

Somewhat

To a great extent

28. How concerned are you about the potential for discrimination based on your patients' genetic test results – with respect to *health insurance*?

Not at all

Very little

Somewhat

To a great extent

29. How prepared do you feel to discuss *health insurance discrimination* concerns with patients?

Not at all

Very little

Somewhat

To a great extent

30. How concerned are you about the potential for discrimination based on your patients' genetic test results – with respect to *life insurance*?

Not at all

Very little

Somewhat

To a great extent

Over →

31. How prepared do you feel to discuss *life insurance discrimination* concerns with patients?

Not at all

Very little

Somewhat

To a great extent

32. How familiar are you with the Genetic Information Non-Discrimination Act (GINA) of 2008?

Not at all

Very little

Somewhat

To a great extent

Background

33. How many years have you been in clinical practice?

0-5

6-10

11-15

16-20

21 or more

34. What percentage of your time is spent in patient care-related activities?

0-10

11-20

21-30

31-40

41-50

51 or more

Over →

35. What is your primary area of practice?

Internal Medicine

Family Medicine

Obstetrics/Gynecology

Other *(please specify):* ___________

36. What is your gender?

Male

Female

Non-binary

Other *(please specify):* ___________

37. Please indicate your age group:

20-29

30-39

40-49

50-59

60-69

70 or older

38. Please select the category that best describes your race/ethnicity:

American Indian or Alaska Native

Asian

Black or African American

Hispanic or Latino

Native Hawaiian or Other Pacific Islander

White

Over →

39. Do you have any other comments?

Thank you for taking time to complete this survey!
